# Characterization of a calcified intra-cardiac pseudocyst of the mitral valve by magnetic resonance imaging including T1 and T2 mapping

**DOI:** 10.1186/1471-2261-14-11

**Published:** 2014-01-28

**Authors:** Ursula Reiter, Gert Reiter, Martin Asslaber, Drago Dacar, Ralph Maderthaner, Josepha Binder, Andreas Greiser, Meinrad Beer, Michael Fuchsjäger

**Affiliations:** 1 Division of General Radiology, Department of Radiology, Medical University of Graz, Auenbruggerplatz 9/P, Graz A-8036, Austria; 2 Siemens AG, Healthcare Sector, Graz, Austria; 3 Institute of Pathology, Medical University of Graz, Graz, Austria; 4 Division of Cardiac Surgery, Department of Surgery, Medical University of Graz, Graz, Austria; 5 Division of Cardiology, Department of Internal Medicine, Medical University of Graz, Graz, Austria; 6 Siemens AG, Healthcare Sector, Erlangen, Germany; 7 Department of Diagnostic and Interventional Radiology, University of Ulm, Ulm, Germany

## Abstract

**Background:**

Even though intra-cardiac cystic lesions are extremely unusual in adults, they should be considered in the differential diagnosis of patients presenting with valvular masses. Cardiac magnetic resonance imaging has emerged as modality of choice for non-invasive characterization of cardiac masses.

**Case presentation:**

We report a case of an intra-cardiac mass of the mitral valve in a 51-year old male, detected by echocardiography after transient ischemic attack and retinal artery occlusion. Cardiac magnetic resonance (CMR) imaging was performed at 3 T to evaluate and characterize the lesion prior to surgery. Diagnosis of a calcified left-ventricular pseudocyst of the mitral valve was confirmed by histological evaluation.

**Conclusions:**

This case presents the unusual finding of contrast uptake in an intra-cardiac cystic lesion and points to the potential of T1 and T2 mapping for assisting in the characterization and diagnosis of intra-cardiac masses by CMR.

## Background

Intra-cardiac cystic lesions are extremely unusual in adults. Classified as benign tumors predominantly involving the cardiac valves and supporting structures, intra-cardiac cysts have been recognized as a cause of intra-cavity flow obstruction, arrhythmia, and valvular dysfunction and have been associated with a risk of embolization [1-3].

Whereas echocardiography is the mainstay imaging technique for the detection of intra-cardiac tumors, multi-parametric cardiac magnetic resonance (CMR) imaging has become the modality of choice for non-invasive characterization of cardiac masses [4]. Comprehensive CMR imaging protocols for the evaluation of cardiac tumors including cine steady-state free precession (SSFP) sequences, black-blood T1- and T2-weighted turbo spin-echo (TSE) imaging with and without fat saturation before and after contrast enhancement, first-pass perfusion and early and late gadolinium enhancement (LGE) have been introduced, providing substantial information on the extent, morphology and vascularization of cardiac lesions [5]. Discrimination of intra-cardiac masses based on image signal intensity patterns, however, remains challenging because of their qualitative nature.

Techniques enabling the quantification of cardiac T1 and T2 magnetic relaxation times within reasonable breath-hold periods [6-9] have yielded remarkable evidence in objective identification of ischemic and non-ischemic myocardial injuries [10-12]. As these magnetic relaxation times provide information about tissue composition on standardized scale (in milliseconds), they may have the potential to further improve the differentiation of cardiac tumors. The application of T1 and T2 mapping for characterization of intra-cardiac masses has not been reported to date.

## Case presentation

**Figure 1:**
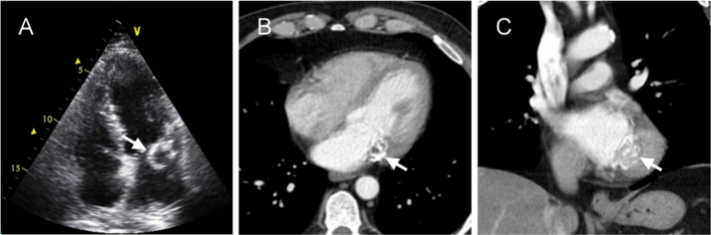
**2D echocardiography and multi-slice CT findings.** Echocardiography **(A)** showed an echolucent mass with a highly echogenic rim (arrow) attached to the mitral valve. Transverse **(B)** and coronal **(C)** chest CT images showed a mass (arrows) adhering to the posterior mitral valve leaflet with peripheral calcification.

A 51-year-old male with a history of smoking underwent transthoracic and transoesophagial echocardiography after a transient ischaemic attack (TIA) and right retinal artery occlusion. Echocardiography documented a mass of 26 mm × 24 mm attached to the mitral valve (Figure 1A), medium degree mitral and tricuspid regurgitation, and thrombosis of the aortic arch and descending aorta. Anticoagulant therapy was immediately initiated. Complete blood count and biochemical tests, including electrolytes, kidney, heart and liver function tests were within normal limits, and repeated cultures of blood and urine were all negative. Thoracic computed tomography (CT) confirmed the presence of a calcified lesion on the mitral valve of unclear extent (Figure 1B,C); no signs of thrombosis of the aorta were found. Based on recurring signs of TIA, including visual disturbance, speech problems and left-side dysaesthesia, the decision was made to excise the lesion surgically.

For pre-surgical evaluation of localization, extend and nature of the mass, the patient was referred for 3 T CMR imaging (Magnetom Trio, Siemens AG, Healthcare Sector, Erlangen, Germany). Differential diagnoses of the mass included caseous calcification of the valvular annulus, valvular calcified thrombus, calcified tumor (calcified myxoma or papillary fibroelastoma of mitral valve), or intra-cardiac cystic lesion. A comprehensive, ECG-gated CMR imaging protocol was carried out in breath-hold and included prototype T1 and T2 mapping sequences.

### CMR imaging

**Figure 2:**
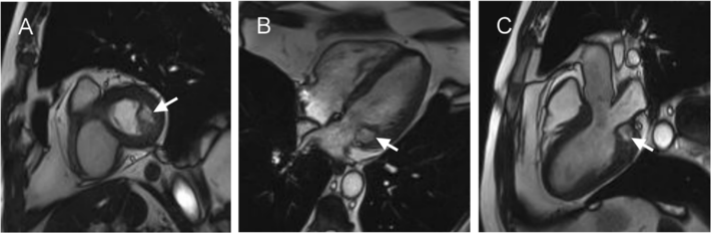
**Diastolic steady-state free-precession MR images of the mass in short-axis (A), 4-chamber (B) and 3-chamber (C) orientations.** The mass (arrows) appears in the left ventricle, with a hyperintense core compared to myocardium and isointense to blood, adherent to the posterior papillary muscle and thickened posterior mitral valve leaflet.

The location and functional characteristics of the lesion were assessed from cine SSFP images covering the entire structure with gapless slices in 4-chamber and short-axis orientations as well as in 3-chamber view (Figure 2 and movie in Additional files 1 and 2). A smooth shaped mass, adhering to the posterior papillary muscle and posterior mitral valve leaflet, which was thickened and restricted in mobility, presented in the left-ventricular cavity, inducing mitral valve regurgitation. Enclosed within a hypointense layer, it appeared hyperintense to myocardium and isointense to blood. Foci of pulsatile signals were observed in the mass, suggesting vascularization. The volume of the lesion, evaluated by manual segmentation in 4-chamber view (Argus, Siemens AG, Healthcare Sector, Erlangen, Germany), however, did not show dependence on cardiac phase (8.6 ml in systole versus 8.4 ml in diastole).

**Figure 3:**
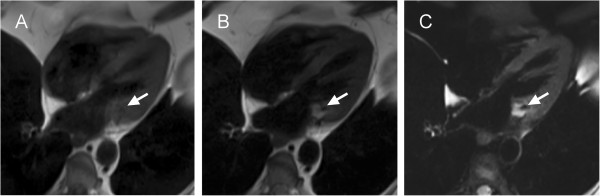
**Turbo spin-echo (TSE) T1-weighted (A), TSE T2-weighted (B) and fat-saturated TSE T2-weighted (C) MR images in 4-chamber orientation.** The mass is isointense to myocardium in the T1-weighted image and displays hyperintense signal on T2-weighted sequences with hypointense regions (arrows).

For standard tissue characterization, dark-blood-prepared T1- and T2-weighted TSE images were acquired in end-diastole. The mass was isointense to myocardium on T1-weighted images (Figure 3A) and hyperintense to myocardium on T2-weighted images (Figure 3B,C). On both, T1-weighted and T2-weighted images, the mass contained hypointense regions indicating either patchy calcification or vascularization.

**Figure 4:**
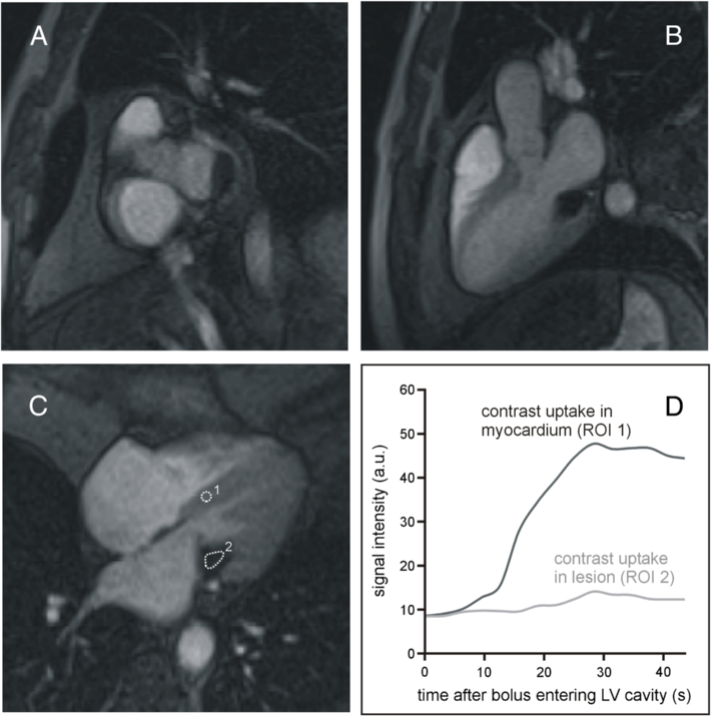
**First-pass perfusion images in short-axis (A), 3-chamber (B) and 4-chamber (C) orientations, along with the signal intensity-time curve (D) of myocardium and the mass.** First-pass images show the mass as hypointense when the contrast agent arrives in the myocardium. Areas 1 and 2 in **(C)** show the regions of interest wherefrom signal intensity-time curves of myocardium (ROI 1) and mass (ROI 2) were derived in 4-chamber view. LV = left ventricle.

Gadolinium was injected at rest as a bolus (Gd-DO3A-butrol, 0.15 mmol per kg body weight followed by 30 ml saline flush, both infused at 3 ml/s). First-pass saturation recovery gradient-echo perfusion images were acquired in three 4-chamber, four short-axis and three 3-chamber slices covering the mass. There were no signs of myocardial infiltration (Figure 4A-C). Semi-quantitative tissue analysis was performed by manually segmenting the lesion in 4-chamber orientation; the results showed low contrast uptake in the lesion (Figure 4D).

**Figure 5:**
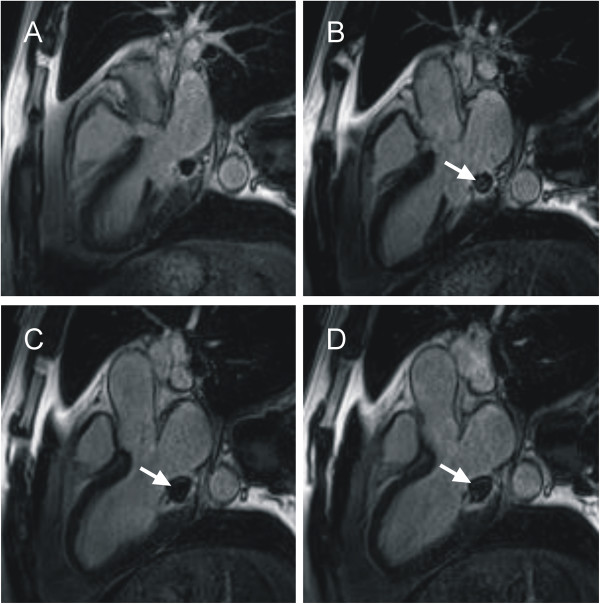
**Late-gadolinium-enhancement imaging.** Images of the lesion in 3-chamber orientation acquired with a three-dimensional inversion recovery FLASH sequence (contiguous slices A through D). Inversion time was chosen to zero the mass. Arrows indicate subtle contrast enhancement in the mass and massive enhancement of the surface.

Post-contrast cine FLASH (fast low-angle shot) imaging performed in 3-chamber, 4-chamber and short-axis orientations revealed the structure as a hypointense mass with foci of pulsatile signals (movie in Additional file 3), confirming findings of vascularization and no infiltration. Imaging of late gadolinium enhancement, acquired by inversion recovery gradient-echo sequences 10 minutes after contrast agent injection, demonstrated a thin, circumferential rim of contrast enhancement around the core of the mass and massive enhancement of the surface in multiple imaging planes (Figure 5).

**Figure 6:**
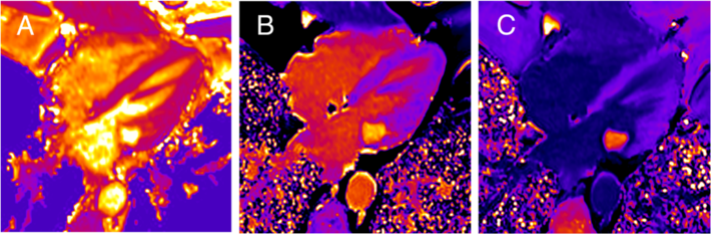
**Pre-contrast T2 map (A) as well as T1 maps obtained before (B) and 15 minutes after (C) contrast agent application in 4-chamber view.** Mean pre-contrast T1, pre-contrast T2 and post-contrast T1 relaxation times of the lesion were 2220 ± 144 ms, 151 ± 17 ms and 925 ± 37 ms, respectively. Mean pre-contrast T1, pre-contrast T2 and post-contrast T1 relaxation times for blood were 1654 ± 47 ms, 147 ± 26 ms, and 271 ± 10 ms, and for myocardium 1144 ± 56 ms, 46 ± 4 ms, and 448 ± 25 ms, respectively.

When all aspects of soft-tissue signal and dynamic characteristics were considered together, the lesion could not be assigned to any of the suspected diagnoses: The morphological findings ruled out caseous calcification of the mitral valve [13] as well as valvular calcified thrombus [5], first-pass gadolinium uptake was inconsistent with the diagnosis of an intra-cardiac cyst [5,14], and the lack of pronounced late gadolinium enhancement excluded the diagnoses of myxoma and papillary fibroelastoma [5,15]. Overall, the findings mainly supported the diagnosis of a calcified intra-cardiac cyst of the mitral valve, because first pass contrast uptake was small and might have been neglected in cases of intra-cardiac cysts reported in literature (Table 1). To further analyse the content and the observed contrast agent uptake of the lesion, pre-contrast T1 and T2 and post-contrast T1 relaxation times were evaluated from single-breath-hold modified Look-Locker inversion recovery (MOLLI) [6,7] and T2 preparation-based T2 mapping [8,9] sequences by manually segmenting the lesion (Figure 6). Mean pre-contrast T1 and T2 times of the lesion of 2220 ± 144 ms and 151 ± 17 ms, respectively. These values were larger than pre-contrast myocardial magnetic relaxation times (T1 = 1144 ± 56 ms, T2 = 46 ± 4 ms), in particular explaining the qualitative pre-contrast signal intensity characteristics of the mass on T1- and T2-weighted TSE as well as SSFP images [16]. Since the T1 time of blood derived from the left ventricular cavity with 1654 ± 47 ms was substantially below T1 of the intra-cardiac lesion, the diagnosis of a blood cyst could be excluded as older blood typically should have shorter T1 times [17]. The decrease of T1 time of the lesion after contrast agent application to 925 ± 37 ms, however, affirmed the observed low contrast agent uptake in first pass perfusion and late enhancement imaging, and so the vascularization of the mass.

**Table 1:** Reported CMR signal intensity characteristics of histologically confirmed intra-cardiac cystic lesions

**Reference**	**Finding**	**SSFP**	**TSE-T1**^**+**^	**TSE-T2**^**+**^	**First pass**	**Late enhancement**
Reichelt et al. [18]	Blood cyst of the papillary muscle	Hyper^+^	-	Hyper	No uptake	No uptake
		Hypo^#^				
Park et al. [15]	Blood cyst of the papillary muscle	Hyper^+^	iso	Hyper	-	No uptake
		Hypo^#^				
Roubelakis et al. [14]	Blood cyst of the tricuspid valve	Hyper^+^	-	-	No uptake	Enhanced border, non-enhanced core
		Hypo^#^				
Centella et al. [19]	Blood cyst in the right atrium	iso^+^	iso	iso	No uptake	No uptake
		Hypo^#^				
Tran et al. [20]	Cystic lesion of the atrio-ventricular node	Hyper^+^	-	-	-	Global enhancement
		Hypo^#^				
Saito et al. [21]	Cystic lesion of the atrio-ventricular node	-	hyper	Hyper	-	-
Shayingca et al. [22]	Cystic lesion of the papillary muscle	Hyper^+^	-	Hyper	-	-
		iso^#^				

SSFP = steady-state free-precession; TSE = turbo spin-echo.

^+^signal compared to normal myocardium; ^#^signal compared to blood, -not reported.

### Surgery and histological diagnosis

**Figure 7:**
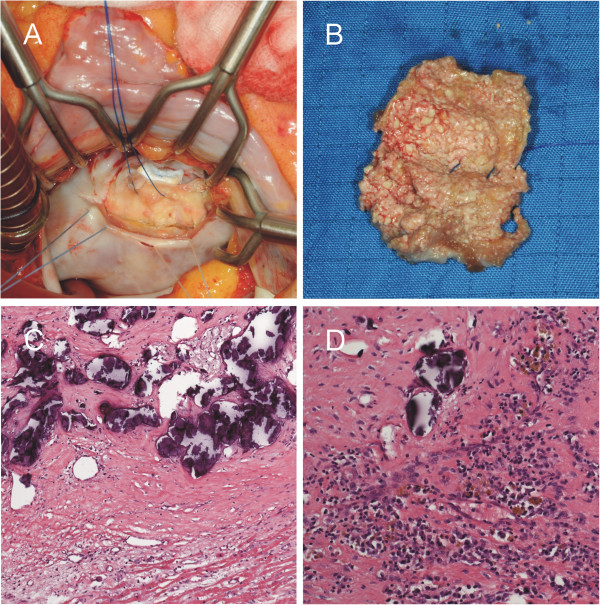
**Surgical (A,B) and histological (C,D) findings.** The smoothly-shaped mass was attached to the mitral valve and the posterior left ventricular wall **(A)**. The inner wall of the lesion consisted of cobblestone-shaped structures **(B)**. Prepared low‒power haematoxylin and eosin (H&E stain) photomicrograph with 10x magnification demonstrated a calcified mass adherent to myocardium **(C)**. 20x H&E stain images of the lesion’s wall showed calcified vessels, residues of blood and regions of chronic inflammation **(D)**.

During surgery a smooth-shaped, white-yellowish mass was found on the ventricular side attached to the mitral valve and the posterior left ventricular wall (Figure 7A). The structure contained a cloudy fluid and presented with a villous inner wall consisting of cobblestone-shaped structures (Figure 7B). The mass was successfully resected and the mitral valve replaced (St. Jude). The postoperative course was uneventful.

Histologic evaluation revealed a calcified pseudocystic mass of the mitral valve. Surrounded by a fibrous, calcified envelope, the wall of the lesion contained a dense layer of connective tissue with multiple occluded vessels and calcifications, without an inner epithelial layer (Figure 7C) and with signs of a chronic inflammatory process indicating chronic endocarditis (Figure 7D). Malignancy was ruled out.

## Conclusions

An intra-cardiac cystic lesion can show contrast agent uptake. CMR enables evaluation of location, size, shape, mobility, and texture of an intra-cardiac lesion. T1 and T2 mapping before and after contrast agent application might provide quantitative information on the nature of a lesion’s content, possibly improving the non-invasive diagnosis and differentiation of intra-cardiac tumors.

### Consent

Written informed consent was obtained from the patient for publication of this case report and any accompanying images. A copy of the written consent is available for review by the Editor of this journal.

## Abbreviations

CMR: Cardiac magnetic resonance; CT: Computed tomography; FLASH: Fast low angle shot; LGE: Late gadolinium enhancement; MOLLI: Modified look-locker inversion recovery; SSFP: Steady-state free-precession; TIA: Transient ischaemic attack; TSE: Turbo spin-echo.

## Competing interests

GR and AG are employed by Siemens Healthcare. All authors declare that they have no conflicts of interest relevant to this manuscript.

## Authors’ contributions

UR and GR drafted the manuscript and acquired and interpreted CMR images. MA performed histology and established the final diagnosis of a calcified pseudocyst of the mitral valve. DD was the surgeon responsible for removing the lesion. RM performed and interpreted chest CT; JB performed and interpreted Echo. AG provided CMR mapping imaging sequences, and MB and MF aided in the analysis and imaging-based diagnosis of the tumor. All authors read and approved the final manuscript.

## Pre-publication history

The pre-publication history for this paper can be accessed here:

http://www.biomedcentral.com/1471-2261/14/11/prepub

## Supplementary Material

Additional file 1 Stack of cine SSFP 4-chamber view slices. Click here for file

Additional file 2 Cine SSFP 3-chamber view images demonstrating focal loci of pulsatile signals suggesting vascularization. Click here for file

Additional file 3 Post-contrast cine FLASH images in 3-chamber orientation. Click here for file
